# Dataset on aquatic ecotoxicity predictions of 2697 chemicals, using three quantitative structure-activity relationship platforms

**DOI:** 10.1016/j.dib.2023.109719

**Published:** 2023-10-24

**Authors:** Patrik Svedberg, Pedro A. Inostroza, Mikael Gustavsson, Erik Kristiansson, Francis Spilsbury, Thomas Backhaus

**Affiliations:** a Department of Biological and Environmental Sciences, University of Gothenburg, PO Box 463, SE-405 30 Gothenburg, Sweden; b Institute for Environmental Research, RWTH Aachen University, D-52072 Aachen, Germany; c Department of Economics, University of Gothenburg, PO Box 640, SE-405 30 Gothenburg, Sweden; d Department of Mathematical Sciences, Chalmers University of Technology and University of Gothenburg, SE-412 96 Gothenburg, Sweden

**Keywords:** Chemical toxicity, Quantitative structure-activity relationship, ECOSAR, Toxicity estimation software tool, VEGA

## Abstract

Empirical and *in silico* data on the aquatic ecotoxicology of 2697 organic chemicals were collected in order to compile a dataset for assessing the predictive power of current Quantitative Structure Activity Relationship (QSAR) models and software platforms. This document presents the dataset and the data pipeline for its creation. Empirical data were collected from the US EPA ECOTOX Knowledgebase (ECOTOX) and the EFSA (European Food Safety Authority) report “Completion of data entry of pesticide ecotoxicology Tier 1 study endpoints in a XML schema – database”. Only data for OECD recommended algae, daphnia and fish species were retained. QSAR toxicity predictions were calculated for each chemical and each of six endpoints using ECOSAR, VEGA and the Toxicity Estimation Software Tool (T.E.S.T.) platforms. Finally, the dataset was amended with SMILES, InChIKey, pKa and logP collected from webchem and PubChem.

Specifications Table

**Table:** 

Subject	Environmental science
Specific subject area	Ecotoxicology and environmental hazard characterisation
Data format	Raw, Filtered
Type of data	Table
Data collection	The empirical data was collected from the ECOTOX database [1] and the EFSA report “Completion of data entry of pesticide ecotoxicology Tier 1 study endpoints in a XML schema – database” [2]. SMILES (Simplified Molecular Input Line Entry System) and InChIKeys (a fixed-length condensed digital representation of the International Chemical Identifier) were collected using the webchem R-package (version 1.1.3) [3] and PubChem [4]. pKa and logP values were also collected from PubChem [4]. QSAR toxicity predictions were generated using the ECOSAR (version 2.2) [5], VEGA (version 1.1.5) [6] and T.E.S.T. (version 5.1.1.0) [7] platforms. All data were filtered, processed and compiled in R (version 4.1.3) using the RStudio environment (version 2022.12.0).
Data source location	US EPA ECOTOX Knowledgebase (ECOTOX) [1] (accessed 15-sep-2022): • https://gaftp.epa.gov/ecotox/ecotox_ascii_09_14_2023.zip Completion of data entry of pesticide ecotoxicology Tier 1 study endpoints in a XML schema – database [2]: • https://doi.org/10.2903/sp.efsa.2012.EN-326 ECOSAR v. 2.2 [5] application available at: • https://www.epa.gov/tsca-screening-tools/ecological-structure-activity-relationships-ecosar-predictive-model VEGA v. 1.1.5 [6] application available at: • https://www.vegahub.eu/portfolio-item/vega-qsar/ T.E.S.T. v. 5.1.1.0 [7] application available at: • https://www.epa.gov/chemical-research/toxicity-estimation-software-tool-test
	Additional secondary data collected (august 2023):
	• Identifiers collected with webchem R-package v. 1.1.3 [3] https://cran.r-project.org/package=webchem • Physicochemical properties collected from PubChem [4] https://pubchem.ncbi.nlm.nih.gov/
Data accessibility	URL to data repository: https://github.com/ThomasBackhausLab/QSAR_predictions_database/tree/main The empirical dataset contains data from the following sources: US EPA ECOTOX [1] ASCII-file direct link: https://gaftp.epa.gov/ecotox/ecotox_ascii_09_14_2023.zip EFSA pesticide report [2]: Pierobon, E., Neri, M. C., Marroncelli, S., & Croce, V. (2012). Completion of data entry of pesticide ecotoxicology Tier 1 study endpoints in a XML schema–database. EFSA Supporting Publications, 9(11), 326E. https://doi.org/10.2903/sp.efsa.2012.EN-326

## 1 Value of the Data


 

• The dataset provides empirical and *in-silico* ecotoxicity data that can be used to benchmark and comparatively assess the predictive performance of different QSAR models for various chemical classes and ecotoxicological endpoints.
• For pesticides, the data allow comparison and analysis of empirical data present in the public domain and with a major regulatory authority (EFSA).
• QSAR predictions require software, knowledge, and time to produce. This dataset provides curated ecotoxicity predictions that are instantly ready to use in the context of chemical hazard and risk characterization.
• This dataset is of use for anyone developing QSAR models, researchers or policy makers interested in the ecotoxicological effects of pesticides, and anyone filling data gaps for chemical risk assessments who might lack the resources to generate large sets of QSAR predictions.
• The standardized format of the dataset makes it easy to combine the generated predictions with the included empirical data, or data from other sources.

## 2 Data Description

The presented dataset includes empirical and predicted ecotoxicological effect data for algae, daphnia, and fish for 2697 organic chemicals. The empirical data has been curated to match the prediction data. Physico-chemical parameters and chemical identifiers are also included.

The data are available in a public GitHub repository [8] that contains the outputs of the data collection (datasets with empirical and predicted ecotoxicity estimates), documentation, links to the primary input data, the secondary input data and all data collection and curation scripts.

### 2.1 Structure of the repository

The root folder of the repository [8] contains the main R-script (“QSAR_data_collection_script.R”), the readme file, the R-project-file and four separate folders (“Additional data”, “Functions”, “Intermediate files” and “Output”).

The “Additional data”-folder contains two files used to process the empirical data, “molweight_lookup.Rda” and “ECOTOX-Term-Appendix-C.csv”. The former is a molecular weight lookup table that is not required to run the main script but improves processing times by reducing the number of queries to the PubChem repository. This file is also appended whenever new molecular weights are retrieved from the PubChem repository. The latter contains a list with the formulation types (types of complex products that contain several chemicals, such as crude oils or fermentation products) for which ecotoxicological data are included in the ECOTOX database. This list is used to filter out those data, as only ecotoxicological data for mono-constituent chemicals are retained in the final dataset.

The “Functions”-folder contains all custom functions used in the main script, such as import, and filter functions and all the functions needed for generating and filtering the QSAR predictions. All individual functions are described in the accompanying readme file.

The “Intermediate files”-folder contains a series of files which are created by the script and then coalesced into the main output file. They are all r-data-files (.Rda) and help to reduce processing times when performing reruns. Loading or overwriting the intermediate files can be controlled with the “rerun” parameter in the main script. Further information on the intermediate files can be found in the readme of the repository and in the main script.

The “Output”-folder contains the empirical and predicted data, which is described below.

### 2.2 The empirical data

The empirical ecotoxicity data that were retrieved from EFSA [1] and the US-EPA [2] for acute and chronic toxicity of defined mono-constituent chemicals to algae, daphnids and fish are included in the TSV-file “experimental_dataset.tsv“, which is located in the “output” folder of the repository. Its structure is summarized in Table 1. The file is presented in long format, with one row for each empirical data point, for a total of 51 954 data points.

**Table 1:** Description of the columns of the empirical data file (“experimental_dataset.tsv”).

Column number range	Naming convention	Description
1-9		Chemical identifiers and test metadata used or collected by the script
10-36	EFSA_*	Test metadata inherited from EFSA
37-227	ECOTOX_*	Test metadata inherited from ECOTOX

*Note:* All columns are described in “QSAR_predictions_database_content_descriptions.xlsx”, located in the “output” folder of the repository [8].

Columns 1 to 9 contain the basic information of the data, either retrieved from the source data or produced by the main analysis script (CAS-numbers, SMILES, Media type, Species name, Endpoint, Duration, concentration, concentration sign and database source).

Columns 10 to 36 contain additional information inherited from the EFSA database (prefixed with EFSA_*), see documentation in [2].

Columns 37 to 227 contain information inherited from the ECOTOX database (prefixed with ECOTOX_*), see the documentation in [1].

### 2.3 The QSAR predictions

The results of the QSAR modeling can be found in the TSV-file “QSAR_predictions.tsv”. It contains at least one QSAR prediction for each of the 2676 chemicals listed. The file is in wide format, i.e., one row per chemical with all QSAR-predictions provided in different columns. A summary of the 589 columns of the file is provided in Table 2. Missing numerical values are encoded as -7777, and missing string values are encoded as “missing”.

**Table 2:** Description of the columns of the main results file (“QSAR_predictions.tsv”).

Column number range	Naming convention	Description
1-8	META_*	Chemical identifiers and physico-chemical data
9-513	ECOSAR_raw_*	Predictions generated by the different ECOSAR models
514-531	ECOSAR_calculated_*	ECOSAR predictions recalculated to a single prediction per endpoint using three different calculation methods: baseline toxicity model (neutral organics) only, geometric mean of all model predictions without considering the baseline toxicity model and the lowest of all model outputs.
532-563	VEGA_raw_*	QSAR estimates generated by the different VEGA modles, each one preceded by the corresponding prediction quality assessment.
564-587	VEGA_calculated_*	VEGA predictions recalculated to a single prediction per endpoint using four different calculation methods: geometric mean of all estimates while excluding low quality predictions and training set data (experimental values); geometric mean of the low quality predictions; the lowest estimate when low quality predictions and experimental values are excluded; the lowest estimate if only low quality predictions are included.
588 and 589	TEST_raw_*	Consensus mode predictions generated by the two different models used in T.E.S.T.

*Note:* All columns are described in “QSAR_predictions_database_content_descriptions.xlsx”, located in the “output” folder of the repository [8].

The first 8 columns are prefixed with META_* and contain identifiers (internal ID, CAS Registration Number, SMILES, InChIKey, PubChem compound ID number (CID)) and physicochemical data (logP, logP source and pKa). The “raw” predictions from the QSAR platforms (prefixed with [PLATFORM]_raw_*) present the outputs from the different QSAR platforms. A series of columns prefixed with [PLATFORM]_calculated* then provides summaries of the QSAR-predictions. The algorithm for calculating these summary columns varies by platform. Details on calculation methods can be found in the materials and methods section.

### 2.4 Chemical identifiers and physico-chemical data

The identifiers and physico-chemical data are collected in the TSV-file “identifiers.tsv“, located in the “Output” folder. This list contains 60 chemicals that are not included in the dataset with QSAR predictions (“QSAR_predictions.tsv”). For these chemicals, empirical data were found in either the EFSA or the ECOTOX database (included in “empirical_dataset.tsv”), but none of the QSAR platforms were able to calculate a toxicity value.

### 2.5 Additional details of the database content

The file “QSAR_predictions_database_content_descriptions.xlsx”, in the “output” folder of the repository, provides details for each column in the files “QSAR_predictions.tsv”, “empirical_dataset.tsv”, and “identifiers.tsv”.

## 3 Experimental Design, Materials and Methods

### 3.1 Compilation and quality check of the empirical data

**Fig. 1:**
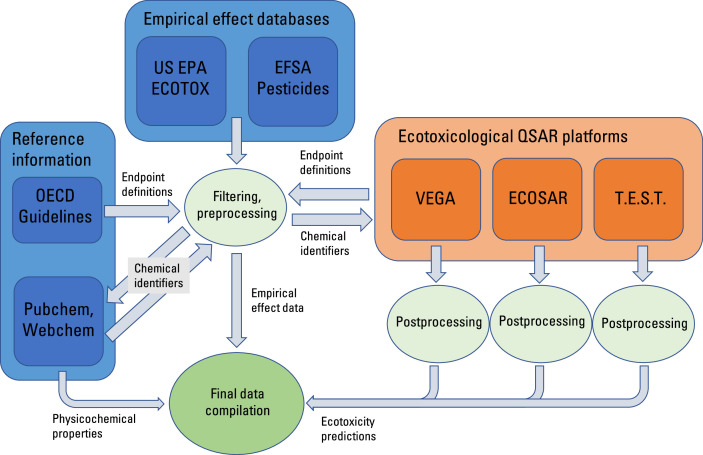
Flowchart of data collection and processing as well as QSAR-modeling. The empirical data originates from the ECOTOX and EFSA databases. OECD guidelines and the endpoints in the QSAR training data defines filter for effect, species and endpoint. Chemical identifiers (SMILES, InChIKey, CID) are collected from PubChem and Webchem using CAS-numbers from the empirical databases. SMILES are entered into the QSAR models, and the output is processed independently (considering the different output formats). The compiled dataset contains the empirical information, the QSAR predictions and the physico-chemical data collected from PubChem.

Empirical data were collected from the European Food Safety Authority (EFSA) [2] and the US Environmental Protection Agency (EPA) [1]. Fig. 1 provides an overview of the collection workflow, including the main filtration and curation steps. Data handling was implemented using R version 4.1.3 [9] using the RStudio editor version 2022.12.0 Build 353 [10]. Table 3 presents all R-packages used.

**Table 3:** Used R-packages and version number.

Package name	Version	Package name	Version
data.table	1.14.2	stringr	1.4.0
Dplyr	1.0.8	tidyr	1.2.0
Readr	2.1.2	tidyverse	1.3.2
Readxl	1.4.0	webchem	1.1.3

### 3.2 Data from EFSA

The EFSA data contains curated ecotoxicological effect data for pesticides originating from dossiers submitted to EFSA under Directive 91/414/EEC [11]. The raw data comprise 33 858 experimental values for 187 pesticides (active ingredients of plant protection products), of which 150 are identified by unique CAS-numbers and 37 are identified by name only. Data curation started with adding the missing CAS-numbers by querying the chemical identifier resolver (CIR) of PubChem [4], using the provided pesticide name, through the webchem package [3]. If no CAS number was retrieved by CIR, CAS names were manually retrieved from PubChem [4]. One mistyped CAS-number (1523233-91-1) was manually replaced by the correct CAS (153233-91-1), identified at PubChem [4]. 17 chemicals (and chemical mixtures) without identifiable CAS-number were discarded.

All species recommended by the considered OECD guidelines were compiled (Table 4), for daphnids see [12,13], for fish see [14,15] and for algae see [16], together with a list of synonyms (i.e. outdated names, homonyms) from the NCBI taxonomic database and AlgaeBase [17,18]. Species names listed in the EFSA database were, if necessary, replaced by the current species name. All test durations were recalculated into hours. SMILES were added using the webchem package [3]. The curated EFSA data were then filtered, using the previously created species lists, to retain only species recommended in the various OECD guidelines (Table 4). Chronic NOECs and EC10 values were assumed equivalent [19] and classified as NOECs in order to match the output format of the various QSAR platforms. For acute endpoints, only EC50/LC50 values were retained for the analysis.

**Table 4:** Empirical data types retained for the analysis.

Test	Endpoint	Species	OECD guideline
Daphnia acute [12]	EC50, LC50	*Daphnia magna*	202
Daphnia chronic [13]	NOEC	*Daphnia pulex*	211
Fish acute [14]	EC50, LC50	*Danio rerio,* *Pimephales promelas,* *Cyprinus carpio,* *Oryzias latipes,* *Poecilia reticulata,* *Lepomis macrochirus,* *Oncorhynchus mykiss*	203
Fish chronic [15]	NOEC		210
Algae acute [16]	EC50, LC50	*Raphidocelis subcapitata,* *Desmodesmus subspicatus*	201
Algae chronic [16]	NOEC		201

Limit values (“larger than” or “smaller than” values) were removed. The EFSA data collection also provides Klimisch reliability scores, which provides an assessment of the reliability of the data [20]. Data with a score of 3 and 4 (“not reliable”, “not assignable”) were removed. Finally, formulations, salts, and entries for marine species were also discarded. After curation and filtering the resulting dataset contains 2 801 individual experimental values from pesticides identified by 148 unique CAS numbers.

### 3.3 Data from ECOTOX

The ECOTOX knowledgebase contains ecotoxicological data, primarily from the open scientific literature, collected by the US EPA [1]. The original files from the ECOTOX database contains 1 154 843 individual experimental values for 12 837 unique CAS numbers. The data were treated similarly to the EFSA data, but with some additional considerations. The downloaded files were first merged into a single table (see ECOTOX_build_function.R for details). The asterisks that note that a recalculation of the concentration was performed before the data were entered into ECOTOX were stripped from the concentration fields. Only the species and endpoints listed in Table 4 were kept. Tests with complex chemical products (e.g., pesticide formulations and petrochemicals) were discarded, together with data from non-freshwater tests. Finally, all limit values were removed.

Concentrations that were reported in molar units were converted to mg/L using molecular weights obtained using webchem [3]. Thereafter, SMILES were added from PubChem [4]. Finally, all salts were removed. The filtered dataset contained 2 673 unique CAS numbers and 49 153 experimental values.

### 3.4 Merging of the empirical data

The column names of the two empirical datasets were, as far as possible, standardized before being merged into a single dataset. The source of each datapoint is documented in a separate column. Chemicals without SMILES were removed and the following identifiers and physicochemical information were added for each chemical: InChIKey and CID from PubChem, collected using the webchem package [3], and logP and pKa from PubChem [4]. A temporary internal ID number was added for each chemical to ensure proper identification when the chemicals were piped into / back from ECOSAR (see below). Finally, SMILES, CAS-numbers and internal IDs were exported for calculating the QSAR predictions. The final empirical dataset contained a total of 51 954 datapoints for 2 757 unique CAS-numbers.

### 3.5 Ecotoxicity predictions by Quantitative Structure Activity Relationships (QSARs)

#### 3.5.1 VEGA platform

SMILES were used to calculate ecotoxicity estimates in VEGA, version 1.1.5 [7] and CAS numbers were used as the chemical identifier. Predictions were produced by all quantitative QSAR models provided by VEGA for daphnids, fish and algae (Table 5). Output was saved in a dedicated summary file, which contains model predictions along with reliability estimates for each prediction, and which was then imported into R. Model names were rewritten into a machine-readable format. The data were transformed from wide format (one row per chemical, multiple columns for the different models) into long format (one row per individual prediction, with one column specifying which model was used to produce a given prediction) to facilitate matching with the other QSAR outputs. Finally, all chemicals for which no QSAR estimate could be calculated were removed from the output file.

**Table 5:** VEGA models for the different species and endpoints.

	Daphnia	Fish	Algae
Acute	Daphnia Magna LC50 48h (EPA) (version 1.0.7) Daphnia Magna LC50 48h (DEMETRA) (version 1.0.4) Daphnia Magna Acute (EC50) Toxicity model (IRFMN) (version 1.0.0) Daphnia Magna Acute (EC50) Toxicity model (IRFMN/Combase) (version 1.0.0)	Fish Acute (LC50) Toxicity model (KNN/Read-Across) (version 1.0.0) Fish Acute (LC50) Toxicity model (NIC) (version 1.0.0) Fish Acute (LC50) Toxicity model (IRFMN) (version 1.0.0) Fish Acute (LC50) Toxicity model (IRFMN/Combase) (version 1.0.0) Fathead Minnow LC50 96h (EPA) (version 1.0.7) Fathead Minnow LC50 model (KNN/IRFMN) (version 1.1.0) Guppy LC50 model (KNN/IRFMN) (version 1.1.0)	Algae Acute (EC50) Toxicity model (IRFMN) (version 1.0.0) Algae Acute (EC50) Toxicity model (ProtoQSAR/Combase) (version 1.0.0)
Chronic	Daphnia Magna Chronic (NOEC) Toxicity model (IRFMN) (version 1.0.0)	Fish Chronic (NOEC) Toxicity model (IRFMN) (version 1.0.0)	Algae Chronic (NOEC) Toxicity model (IRFMN) (version 1.0.0)

*Note:* Further details for the VEGA model platform can be found in [6], and at vegahub.eu

#### 3.5.2 ECOSAR platform

For ECOSAR version 2.2 [3], the input SMILES list was first split into multiple smaller lists in order to reduce runtimes. Each file was run separately, and all ECOSAR output files were merged prior to the import into the main results file. If ECOSAR did not compute QSAR estimates using SMILES, as second attempt was made using CAS-numbers.

In a few cases, ECOSAR identifies multiple possible chemicals for a single SMILES input, and to identify which chemical matches the original CAS numbers from the database, input and output CAS numbers were compared. If there still was uncertainty, metals in the SMILES were compared, since ECOSAR sometimes suggests multiple different metals if there is a metal in the input SMILES. As a third option complete SMILES were compared. One chemical (CAS 128-10-9) was manually identified. All model names were rewritten into a machine-readable format. Chemicals with molecular weight of more than 1000 g/mol or exceeding the upper logP applicability domain limit were removed. All predictions for saltwater organisms were also removed, and finally empty predictions were deleted from the output file.

#### 3.5.3 T.E.S.T. platform

The Toxicity Estimation Software Tool (T.E.S.T.) [4], version 5.1.1.0, was loaded with SMILES and run in batch mode with the *Daphnia magna* LC50 consensus model and *Pimephales promelas* LC50 consensus model. The T.E.S.T. platform does not include any model for estimating algal toxicity or any chronic toxicity. Model names were translated into a machine-readable format and predictions without numerical results were removed.

#### 3.5.4 Final processing

The outputs from VEGA, ECOSAR and T.E.S.T. were imported into R and merged. For this purpose, the field names of all QSAR predictions were standardized and a QSAR-platform identifier field was added. For ECOSAR and VEGA, summary predictions were calculated for each chemical and endpoint, which were tagged with “calculated”. For ECOSAR the “calculated”-columns contain the lowest estimate or the geometric mean of all model estimates per chemical, excluding the baseline-toxicity model for neutral organics (tagged with “no baseline”). If only the model for neutral organics provided a prediction for a given chemical the column is tagged with “only baseline”. In these cases, the lowest value and the geometric mean are thus identical. The VEGA platform provides a quality score for each prediction (an assessment of applicability for each model-chemical combination), which is reported in a separate column. Consequently, the summary columns that present the VEGA-calculated estimates contain the lowest QSAR estimate or the geometric mean and are tagged with “no low no exp” if low quality predictions and experimental values are removed and with “no moderate no good no exp” if moderate and good quality predictions and experimental values are removed. For T.E.S.T. there are no summary columns, as the T.E.S.T.-consensus model provides only one value per chemical and endpoint. The predictions from the QSAR platforms were labelled with “raw”.

Finally, the prediction dataset was exported as “QSAR_predictions.tsv”. The final prediction dataset contained 144 573 individual raw and calculated predictions for 2697 unique CAS-numbers

## Limitations

CAS-numbers were used to identify all chemicals, whereas SMILES were used as input for running the QSAR predictions. The translation from CAS to SMILES, implemented via the webchem package, may have introduced misidentifications of chemicals and isomers. Furthermore, the empirical data were taken at face value, no additional quality or reliability check beyond the Klimisch score filtering of the EFSA data was performed. Finally, in contrast to VEGA and ECOSAR, the T.E.S.T. QSAR platform includes neither models for chronic endpoints for daphnia and fish, nor any algal models.

## Ethics Statement

The authors have read and follow the ethical requirements for publication in Data in Brief and confirm that the current work does not involve human subjects, animal experiments, or any data collected from social media platforms.

## CRediT authorship contribution statement

**Patrik Svedberg:** Conceptualization, Methodology, Investigation, Software, Data curation, Writing – original draft, Visualization. **Pedro A. Inostroza:** Conceptualization, Writing – review & editing, Supervision. **Mikael Gustavsson:** Conceptualization, Writing – review & editing. **Erik Kristiansson:** Conceptualization. **Francis Spilsbury:** Software. **Thomas Backhaus:** Conceptualization, Software, Writing – review & editing, Supervision.

## Data Availability

QSAR predictions database (Original data) (GitHub)
